# Supplementary material for: Changes of bioactive components and antioxidant potential during fruit development of Prunus humilis Bunge
Source: PLoS One. 2021 May 19;16(5):e0251300. doi: 10.1371/journal.pone.0251300 (PMC8133403; doi:10.1371/journal.pone.0251300)
Supplement: Attachment — Submitted filename: Response to reviewer.doc [file pone.0251300.s002.doc]

Thank you for your comments, and I will list my changes as below:

1. English language usage should be substantially polished.

**Reply**: About our English writing, we would like to thank Editage (www.editage.com) for English language editing. And we have marked the changes in **blue** font throughout the manuscript.

2. Figure captions should be completely rewritten in order to be self-explanatory.

**Reply**: The captions of Figure 1-5 had be completely rewritten. Figure 1 we added, Note:The same color letters indicate significance at *P*<0.05. FW, Fresh weight; YF, Young fruit stage; SH, Stone harding stage; ES, Expension stage; TS, Turning stage; CS, Coloring stage; RS, Ripening stage (page 10, line 215-217). Figure 2 we added, Note:The same color letters indicate significance at *P*<0.05. FW, Fresh weight; YF, Young fruit stage; SH, Stone harding stage; ES, Expension stage; TS, Turning stage; CS, Coloring stage; RS, Ripening stage (page 11, line 234-236). Figure 3 we added, Note:The same color letters indicate significance at *P*<0.05. FW, Fresh weight; TE, Trolox equivalent; DPPH, 2,2-diphenyl-1-picrylhydrazyl free radical scavenging capacity; YF, Young fruit stage; SH, Stone harding stage; ES, Expension stage; TS, Turning stage; CS, Coloring stage; RS, Ripening stage (page 12, line 252-255). Figure 4 we added, Note:The same color letters indicate significance at *P*<0.05. FW, Fresh weight; TE, Trolox equivalent; FRAP, ferric reducing antioxidant power; YF, Young fruit stage; SH, Stone harding stage; ES, Expension stage; TS, Turning stage; CS, Coloring stage; RS, Ripening stage (page 13, line 268-271). Figure 5 we added, Note:The same color letters indicate significance at *P*<0.05. FW, Fresh weight; TE, Trolox equivalent; ABTS, 2,2’-azinobis (3-ethylbenzothiazoline-6-sulfonic acid) free radical scavenging capacity; YF, Young fruit stage; SH, Stone harding stage; ES, Expension stage; TS, Turning stage; CS, Coloring stage; RS, Ripening stage (page 14, line 282-285).

3. Statistical significance must be introduced in Figures 1-5.

**Reply**: We analyzed the statistical significance and introduced in Figures 1-5.

4. Please put labels of statistical significance in Table 2 into superscript.

**Reply**: We have already put labels of statistical significance in Table 2 into superscript, and marked in **blue** font.

5. Do not use the entire Latin name "Prunus humilis" throughout the text, but instead "P. humilis". However, "Prunus humilis" should stand in the first mention in the main title, Abstract, and Introduction section. It also applies to other species' Latin names in the text, such as "Camellia japonica".

**Reply**: We have used "*P. humilis*" instead "*Prunus humilis*" throughout the text the except in the first mention in the main title, Abstract, and Introduction section and also applies to other species and marked in **blue** font. And we have maked some changes as below and has marked in blue font in the manuscript:

1) Page 4, line 88; Page 5, line 97; Page 10, line 201; Page 26, line 482; Page 27, line 495, 499, 504; *Prunus humilis* was changed with *P. humilis*

2) Page 26, line 492, *Camellia japonica* was changed with *C. japonica*

6. Please precise what Nongda 4, Nongda 5, 02-16 and DS-1 represent. If they are cultivars, then they should stand under single quotation marks as 'Nongda4' and so on.
**Reply**: Nongda 4, Nongda 5, 02-16 and DS-1 were all cultivars, we standed under single quotation marks as ‘Nongda4’ and marked in **blue** font throughout the manuscript.

1. The title needs edits to reflect "the changes are during fruit development".

**Reply**: The title Dynamic changes of bioactive components and antioxidant potential of *Prunus humilis* Bunge fruit was changes with Changes of bioactive components and antioxidant potential during fruit development of *Prunus humilis* Bunge, and marked in **blue** font

2. Taking into account the amount of results obtained and the different varieties used, perhaps, the article should be structured in a different way. For example, a Principal component analysis (PCA) could be conducted to see the overall differences in phenolic profile and antioxidant activities during different development stages, according to the variety studied (i.g., TPC, TFC, DPPH, FRAP, ABTS, and individual compounds).

**Reply**: We have already added the Principal component analysis(page 21-22, line 386-407),and marked in **blue** font

PCA results identified two components that explained 82.09% of the total variation in bioactive components and antioxidant potential during the fruit development stage among the four *P. humilis* accessions (Fig 7). The first principal component (Prin1) contributed to 72% of the total variation. The second principal component (Prin2) contributed to 9.45% of the total variation.

Scatterplots from the PCA showed that three circles were drawn (Fig 7), and the first three development stages of ‘DS-1’ were brought together separately because the second principal component was lower. The first four stages of ‘02-16’, the first three stages of ‘Nongda 4’, and the first two stages of ‘Nongda 5’ were classified into the same circle, owing to the higher components of Prin 1 and 2. With the development of fruit, the bioactive components and antioxidant potential decreased gradually, and the last two development stages of the four accessions were classified into one circle. These data suggest that from the expension stage to the turning stage, the content of total flavonoids, total phenols, individual compounds, and antioxidant indices showed a tipping point of decline.

Fig 7. Scatterplot of principal component analysis based on bioactive components and antioxidant potential during fruit development of Prunus humilis.

Note: YF, Young fruit stage; SH, Stone hardening stage; ES, Expension stage; TS, Turning stage; CS, Coloring stage; RS, Ripening stage. The three circles indicate the development stages belonging to the top two principal components.

3. Spell out the abbreviation FW for the first time, and note all the abbreviations in the legend for Figure 1-5.

**Reply**: The abbreviation FW for the first time was spell out at page 10, line 204, The flavonoid content in ‘nongda 5’ fruit was keep decreasing from the young fruit stage (56.45 mg/g FW, fresh weight).

All the abbreviations in the legend for Figure 1-5. Figure 1 we added, Note:The same color letters indicate significance at *P*<0.05. FW, Fresh weight; YF, Young fruit stage; SH, Stone harding stage; ES, Expension stage; TS, Turning stage; CS, Coloring stage; RS, Ripening stage (page 10, line 215-217). Figure 2 we added, Note:The same color letters indicate significance at *P*<0.05. FW, Fresh weight; YF, Young fruit stage; SH, Stone harding stage; ES, Expension stage; TS, Turning stage; CS, Coloring stage; RS, Ripening stage (page 11, line 234-236). Figure 3 we added, Note:The same color letters indicate significance at *P*<0.05. FW, Fresh weight; TE, Trolox equivalent; DPPH, 2,2-diphenyl-1-picrylhydrazyl free radical scavenging capacity; YF, Young fruit stage; SH, Stone harding stage; ES, Expension stage; TS, Turning stage; CS, Coloring stage; RS, Ripening stage (page 12, line 252-255). Figure 4 we added, Note:The same color letters indicate significance at *P*<0.05. FW, Fresh weight; TE, Trolox equivalent; FRAP, ferric reducing antioxidant power; YF, Young fruit stage; SH, Stone harding stage; ES, Expension stage; TS, Turning stage; CS, Coloring stage; RS, Ripening stage (page 13, line 268-271). Figure 5 we added, Note:The same color letters indicate significance at *P*<0.05. FW, Fresh weight; TE, Trolox equivalent; ABTS, 2,2’-azinobis (3-ethylbenzothiazoline-6-sulfonic acid) free radical scavenging capacity; YF, Young fruit stage; SH, Stone harding stage; ES, Expension stage; TS, Turning stage; CS, Coloring stage; RS, Ripening stage (page 14, line 282-285).

4. Table 1 indicate the year.

**Reply**: Table 1 indicate the year, we changed the Table 1 Sampling date of *Prunus humilis* during fruit development stage with Table 1 Sampling date of *Prunus humilis* during fruit development stage in 2019. We also indicate the year in plant materials after were acquired for per accession we added in 2019.

5. Line 202 “…showed a decrease in…”

**Reply**: showed a decreased with was changes with showed a decrease in (page 10, line 201)

6. It looks like no significant analysis used for illustrating the results of Figure 1-5, and only mean values were compared.

**Reply**: We analyzed the statistical significance and introduced in Figures 1-5.

7. Line 238-239 Rephrase this sentence.

**Reply**: Among the four accessions nongda 5 had a low DPPH scavenging ability level and 02-16 had a strong DPPH scavenging ability level at all the fruit development stages. Changes with At all fruit development stages, ‘Nongda 5’ had a low DPPH scavenging ability level, and ‘02-16’ had a strong DPPH scavenging ability level. and marked in **blue** font.

1. I advise the authors to find a native English speaker to proofread the manuscript.
**Reply**: About our English writing, we would like to thank Editage (www.editage.com) for English language editing.. And we have marked the changes in **blue** font throughout the manuscript.

2. The abstract should be rewritten. A brief description of the work with the most relevant results should be given. Thus, readers can get an idea of the content of the work.

**Reply**: The abstract has been rewritten, Dynamic changes in flavonoid, total phenol, and antioxidant potential in different *Prunus humilis* accessions during fruit development stages were studied in order to provide a reference for the optimum harvest time for flavonoid extraction. ‘Nongda 4’, ‘Nongda 5’, ‘DS-1’ and ‘02-16’ were selected as plant materials to determine the content of flavonoid, total phenol and antioxidant indices during six fruit development stages. Changes in total flavonoid content (TFC) and total phenol content (TPC) in different accessions of *P. humilis* were slightly different depending on the development stage of *P. humilis* fruit. TFC and TPC in ‘Nongda 5’ fruit showed a trend of continuous decline. There was a small increase in TFC and TPC from the young fruit stage to the stone hardening stage, followed by a decreasing trend, and then to the lowest level at the ripening stage of ‘Nongda 4’, ‘DS-1’, and ‘02-16’ fruits. The trend of antioxidant capacity (ABTS, FRAP, DPPH) with the TFC and TPC of *P. humilis* fruit was basically the same, and the correlation analysis results showed that the TFC of *P. humilis* fruit was positively correlated with the antioxidant indices (P<0.01). Catechin (CC), rutin (RT), and quercetin-7-O-β-D-glucopyranoside (Q7G) were detected in all the fruit development stages of the four *P. humilis* fruits. Among them, catechin was the most abundant component, accounting for approximately 10%. Myricetin (MC) and quercetin (QC) were generally detected only in the early fruit development stage, but not in the later fruit development stage. Correlation analysis showed that the flavonoid components with TFC, TPC, and antioxidant indices differed between the different accessions. RT, CC, and liquiritigenin (LR) had a stronger correlation with TFC and antioxidant indices. Cyanidin-3-O-glucoside (C3G) was not detected until the coloring stage in two red *P. humilis* accessions (‘Nongda 4’ and ‘DS-1’), and so it is better to choose a red *P. humilis* fruit to extract C3G at the ripening stage. Selecting an early stage of fruit development, especially the stone hardening stage, was important for extracting flavonoids, total phenols and other components. We believe that our results will provide basic information and reference for evaluation of fruit nutrition and health benefits, breeding of functional new varieties, and efficient utilization of *P. humilis* fruit.

3. Introduction. The sentences in Lines 82-84 and 84-87 are redundant.
**Reply**: The sentences in Lines 82-84 and 84-87 was delete and written as Flavonoids benefit the human body due to their antioxidant and antiaging properties. Although the total flavonoid content, polyphenol compounds, and radical scavenging activity of *P. humilis* in Shanxi province [6,7], Liaoning province [8], Beijing city [3] and other places [9] have been evaluated, fruit was evaluated only at the ripening stage of development.

4. I suggest separating plant material and chemicals into different sections.

**Reply**: We separated plant material and chemicals into two sections.

5. Authors should include how they expressed the different results. In reference to the replicates. Please change lines 104-105 to "A total of three biological replicated consisting in 3 healthy plants each one was selected” or something like that.

**Reply**: Three healthy plants were selected per accession as a repeated, a total of repeated thrice was changed with A total of three biological replicated consisting in 3 healthy plants each one was selected (Page 5, line 101-102)

6. Authors should include how they expressed TFC, TPC, antioxidant capacity determined by the the three methods.

**Reply**: We added the expressed TFC, TPC, antioxidant capacity determined by the the three methods. Total flavonoid content (TFC)=C_1_×(10/V_1_)×V_2_×m^-1^, total phenol content (TPC)=C_2_×(8/V_1_)×V_2_×m^-1^, antioxidant indices (DPPH, FRAP, and ABTS)=C_3_×V_1_×V_2_×m^-1^×1000. C_1_ indicates the concentration of rutin, C_2_ of gallic acid, C_3_ of trolox, V_1_ the volume of reaction solution, V_2_ the volume of extraction solution, and m the fruit weight. (page 9, line 183-187).

7. Flavonoid content: Please indicate at which range were performed the different calibration curves. Moreover, which software were used for the acquisition data?
**Reply**: Rutin was used as standard curve ranged from 0 mg/ml to 0.1 mg/ml (page 6, line 130-131). Data were analyzed in Microsoft Excel v. 2007 (Microsoft Corporation, Redmond, WA, USA).

8. This section should be rewritten. Moreover, the Figure legends should be improved because Authors did not include any reference to the results showed in each one (biological samples, statiscal method, etc). They only included the titles. Furthermore, the statistical results should be included in the Figures. Based on the results showed it is difficult to know whether some of the changes are statistically different.
**Reply**: We analyzed the statistical significance and introduced in Figures 1-5. The reference of Figure 1-5 had be completely. Figure 1 we added, Note:The same color letters indicate significance at *P*<0.05. FW, Fresh weight; YF, Young fruit stage; SH, Stone harding stage; ES, Expension stage; TS, Turning stage; CS, Coloring stage; RS, Ripening stage (page 10, line 215-217). Figure 2 we added, Note:The same color letters indicate significance at *P*<0.05. FW, Fresh weight; YF, Young fruit stage; SH, Stone harding stage; ES, Expension stage; TS, Turning stage; CS, Coloring stage; RS, Ripening stage (page 11, line 234-236). Figure 3 we added, Note:The same color letters indicate significance at *P*<0.05. FW, Fresh weight; TE, Trolox equivalent; DPPH, 2,2-diphenyl-1-picrylhydrazyl free radical scavenging capacity; YF, Young fruit stage; SH, Stone harding stage; ES, Expension stage; TS, Turning stage; CS, Coloring stage; RS, Ripening stage (page 12, line 252-255). Figure 4 we added, Note:The same color letters indicate significance at *P*<0.05. FW, Fresh weight; TE, Trolox equivalent; FRAP, ferric reducing antioxidant power; YF, Young fruit stage; SH, Stone harding stage; ES, Expension stage; TS, Turning stage; CS, Coloring stage; RS, Ripening stage (page 13, line 268-271). Figure 5 we added, Note:The same color letters indicate significance at *P*<0.05. FW, Fresh weight; TE, Trolox equivalent; ABTS, 2,2’-azinobis (3-ethylbenzothiazoline-6-sulfonic acid) free radical scavenging capacity; YF, Young fruit stage; SH, Stone harding stage; ES, Expension stage; TS, Turning stage; CS, Coloring stage; RS, Ripening stage (page 14, line 282-285).

9. Lines 289-291. Please delete. Authors should only refer to the results of this section. Such sentences should be in the discussion or in the results section where correlations are discussed.

**Reply**: Lines 289-291 has been deleted

10. Lines 315-321. Please move to discussion section.

**Reply**: We moved lines 315-321 after the sentence,it plays an important role in the antioxidant potential

11. In reference to the results included in the correlation analysis, Authors should avoid repeating that "the level of correlation is different". What is important is the biological sense of that correlation, not whether it was 0.99 or 0.93.
**Reply**: The sentence also the correlation coefficient was different has been deleted. The results included in the correlation analysis has been rewritten. The biological sense of that correlation has been written in discussion (page25-26, line458-477), and marked in **blue** font. There were differences in flavonoid content during the fruit development stages among the four accessions determined in this study, CC, rutin, and Q7G were detected in all fruit development stages of the four *P. humilis* fruits. Myricetin and Quercetin were detected in the early developmental stages of ‘Nongda 4’, ‘Nongda 5’, and ‘02-16’, but were not detected in the late developmental stage. C3G was detected in the late developmental stages of ‘Nongda 4’ and ‘DS-1’ , but not detected in any of the developmental stages of ‘Nongda 5’ and ‘02-16’. Correlation analysis showed that there were some differences in the correlation between flavonoid components and total flavonoid content with antioxidant indices among different accessions. Rutin, CC and LR were correlated with flavonoid content and antioxidant indices. Q7G and EC were not significantly correlated with flavonoid content or antioxidant indices. CC, EC, and RT were the main flavonoid components in the fruits of six Chinese jujube cultivars, and they were positively correlated with flavonoid content and DPPH free radical scavenging activity; these three components are the most important antioxidants in Chinese jujube fruit [13]. During fruit development, the flavonoid content in the peel and pulp of Nanfeng tangerine decreased gradually; among the 11 flavonoid components, eight were detected in the peel and four in the pulp, which was not consistent with the observed changes in flavonoid content [19]. There are differences in flavonoid components among different species, but there is obvious correlation between flavonoid content and antioxidant indices.

12. Lines 337-339. Please delete.

**Reply**: Lines 337-339 has been deleted.
